# Synthetic Analogues of Huwentoxin-IV Spider Peptide With Altered Human Na_V_1.7/Na_V_1.6 Selectivity Ratios

**DOI:** 10.3389/fcell.2021.798588

**Published:** 2021-12-20

**Authors:** Ludivine Lopez, Jérôme Montnach, Barbara Oliveira-Mendes, Kuldip Khakh, Baptiste Thomas, Sophia Lin, Cécile Caumes, Steven Wesolowski, Sébastien Nicolas, Denis Servent, Charles Cohen, Rémy Béroud, Evelyne Benoit, Michel De Waard

**Affiliations:** ^1^ L’institut du Thorax, INSERM, CNRS, UNIV NANTES, Nantes, France; ^2^ Xenon Pharmaceuticals, Burnaby, BC, Canada; ^3^ Smartox Biotechnology, Saint-Egrève, France; ^4^ Département Médicaments et Technologies pour La Santé (DMTS), Service d’Ingénierie Moléculaire pour La Santé (SIMoS), ERL CNRS/CEA, Institut des Sciences du Vivant Frédéric Joliot, CEA, Université Paris Saclay, Gif-sur-Yvette, France; ^5^ LabEx « Ion Channels, Science and Therapeutics », Valbonne, France

**Keywords:** huwentoxin-IV analogues, *Cyriopagopus schmidti*, development of pain therapeutics, Na_V_1.6 and Na_V_1.7 channel subtypes, automated patch-clamp, structure-function relationship, peptide synthesis

## Abstract

Huwentoxin-IV (HwTx-IV), a peptide discovered in the venom of the Chinese bird spider *Cyriopagopus schmidti*, has been reported to be a potent antinociceptive compound due to its action on the genetically-validated Na_V_1.7 pain target. Using this peptide for antinociceptive applications *in vivo* suffers from one major drawback, namely its negative impact on the neuromuscular system. Although studied only recently, this effect appears to be due to an interaction between the peptide and the Na_V_1.6 channel subtype located at the presynaptic level. The aim of this work was to investigate how HwTx-IV could be modified in order to alter the original human (h) Na_V_1.7/Na_V_1.6 selectivity ratio of 23. Nineteen HwTx-IV analogues were chemically synthesized and tested for their blocking effects on the Na^+^ currents flowing through these two channel subtypes stably expressed in cell lines. Dose-response curves for these analogues were generated, thanks to the use of an automated patch-clamp system. Several key amino acid positions were targeted owing to the information provided by earlier structure-activity relationship (SAR) studies. Among the analogues tested, the potency of HwTx-IV E^4^K was significantly improved for hNa_V_1.6, leading to a decreased hNa_V_1.7/hNa_V_1.6 selectivity ratio (close to 1). Similar decreased selectivity ratios, but with increased potency for both subtypes, were observed for HwTx-IV analogues that combine a substitution at position 4 with a modification of amino acid 1 or 26 (HwTx-IV E^1^G/E^4^G and HwTx-IV E^4^K/R^26^Q). In contrast, increased selectivity ratios (>46) were obtained if the E^4^K mutation was combined to an additional double substitution (*R* ^26^A/Y^33^W) or simply by further substituting the C-terminal amidation of the peptide by a carboxylated motif, linked to a marked loss of potency on hNa_V_1.6 in this latter case. These results demonstrate that it is possible to significantly modulate the selectivity ratio for these two channel subtypes in order to improve the potency of a given analogue for hNa_V_1.6 and/or hNa_V_1.7 subtypes. In addition, selective analogues for hNa_V_1.7, possessing better safety profiles, were produced to limit neuromuscular impairments.

## Introduction

For over 10 years, the Na_V_1.7 channel subtype is considered as an attractive pain target owing to several compelling genetic evidences. Loss-of-function mutations in *SCN9A*, the gene encoding human (h) Na_V_1.7, lead to congenital insensitivity to pain (referred to as CIP) (Cox et al., 2006). Conversely, gain-of-function mutations of the same gene lead to erythromelalgia and paroxysmal extreme pain disorder (Estacion et al., 2008). Because of these clinical evidences, the hNa_V_1.7 channel has been the target of choice for high-throughput screening campaigns to identify blockers as pain therapeutics. Two types of libraries were used for this purpose: small organic compounds from leading pharmaceutical companies (Macsari et al., 2012; Nguyen et al., 2012; Sun et al., 2014; Focken et al., 2016; Wu et al., 2017; Cuesta and Meneses, 2021) and natural peptides originating from animal venoms (Trim et al., 2021). Indeed, disulfide-bridged peptides, frequently purified from animal venoms, have been considered as interesting lead compounds (Bordon et al., 2020), either as pore blockers or gating modifiers, for the modulation of ion channels in general and the treatment of pain in particular (Cardoso and Lewis, 2018). Owing to chemical spaces larger than those of small molecules, they possess better affinities and selectivity profiles even when the target of interest shares high sequence identity with other ion channel subtypes. This was illustrated by peptides issued from spider venoms, often possessing an Inhibitory Cystine Knot (ICK) fold, that were shown to be able of distinguishing closely related subtypes of Na_V_ channels (for reviews, see (Gonçalves et al., 2018a; Cardoso and Lewis, 2019; Dongol et al., 2019)). This ability is facilitated by the interaction of these peptides with the different voltage-sensor domains, known to be the most divergent in sequence in contrast to pore region.

Along with protoxin II (Montnach et al., 2021), one of the best studied inhibitors of hNa_V_1.7 channel is huwentoxin-IV (HwTx-IV), a 35 amino acid peptide isolated from the venom of the Chinese bird-eating tarantula spider *Cyriopagopus schmidti* (Peng et al., 2002). HwTx-IV belongs to the NaSpTx family 1 (Klint et al., 2012) because of its sequence. In addition, according to the ICK motif, this peptide is folded with the Cys^2^-Cys^17^, Cys^9^-Cys^24^, Cys^16^-Cys^31^ disulfide bridge pattern, which favors the formation of a double-stranded antiparallel beta-sheet (Leu^22^-Ser^25^, Trp^30^-Tyr^33^) along with four turns (Glu^4^-Lys^7^, Pro^11^-Asp^14^, Lys^18^-Lys^21^, Arg^26^-Arg^29^). Overall, this peptide possesses a compact and rigid scaffold (Peng et al., 2002) that confers high protease resistance and thus elevated *in vivo* stability. Like most other spider toxins, HwTx-IV has been reported to be a gating modifier by interacting with the voltage-sensor domain II of hNa_V_1.7 and trapping it into the closed configuration (Xiao et al., 2008; Xiao et al., 2011; Shen et al., 2019; Gao et al., 2020). This binding was suggested to imply residue Glu^753^ of the S1-S2 loop and four residues, Glu^811^, Leu^814^, Asp^816^ and Glu^819^, of the S3-S4 loop of this channel subtype, which defines an EELDE motif for the toxin interaction. Because of the nature of this complex interaction, HwTx-IV owns *de facto* good natural selectivity for hNa_V_1.7. However, hNa_V_1.1, hNa_V_1.2, hNa_V_1.3 and hNa_V_1.6 channel subtypes are also sensitive to the peptide, while hNa_V_1.4, hNa_V_1.5 and hNa_V_1.8 are resistant (Rahnama et al., 2017; Gonçalves et al., 2018b). Indeed, the EELDE motif for HwTx-IV binding onto hNa_V_1.7 is preserved in hNa_V_1.6, and only slightly modified (towards an EELNE motif) in hNa_V_1.1, hNa_V_1.2 and hNa_V_1.3. Many more alterations in this motif are detected in hNa_V_1.4, hNa_V_1.5 and hNa_V_1.8. These observations are all coherent with the selectivity data (Xiao et al., 2008; Revell et al., 2013; Rahnama et al., 2017; Gonçalves et al., 2018b; Agwa et al., 2020).

Considering the high potency of HwTx-IV for hNa_V_1.7, it was quite logical to test the analgesic potential of the peptide in pain animal models. HwTx-IV was thus reported as an efficient analgesic in rodent models of inflammatory and neuropathic pain (Liu et al., 2014b), as well as of spontaneous pain induced by the Na_V_1.7 activator OD1, the first toxin isolated from the venom of the scorpion *Odonthobuthus doriae* (Rahnama et al., 2017). However, these encouraging preclinical results hampered clinical development because the peptide also produced evident side effects *in vivo* resulting from impairment of neuromuscular transmission (Liu et al., 2014a; Deuis et al., 2016; Flinspach et al., 2017; Rahnama et al., 2017). It is only later that this effect was clearly attributed to the activity of HwTx-IV on the Na_V_1.6 channel, a subtype localized at presynaptic nerve terminals innervating the muscles (Gonçalves et al., 2018b).

Because of the analgesic potential of HwTx-IV, mainstream efforts have been dedicated to producing analogues with enhanced potency on hNa_V_1.7 (Revell et al., 2013; Rahnama et al., 2017). However, most of earlier studies, if not all, considered the potential effects of the peptide sequence modifications on its selectivity profile towards other Na_V_ subtypes (in general Na_V_1.5 or Na_V_1.2) with the notable exception of the Na_V_1.6 subtype (Minassian et al., 2013; Neff et al., 2020). The present report aims at investigating the relative selectivity of HwTx-IV and 19 synthetic analogues on both hNa_V_1.7 and hNa_V_1.6 channel subtypes. All these analogues were designed on the basis of earlier SAR investigations and mainly by focusing on amino acid residues shown to influence the hNa_V_1.7/hNa_V_1.2 selectivity ratio. Two goals were pursued: 1) identify analogues with improved selectivity for hNa_V_1.7, and 2) identify new analogues that have improved potencies for hNa_V_1.6. For this purpose, similar experimental conditions for chemical syntheses and functional evaluations were employed to get reliable and exploitable results. Under these conditions, 11 analogues with increased apparent affinity for hNa_V_1.6 were identified, among which 4 improved the half maximal inhibitory concentration (IC_50_) values by more than 36-fold without drastically decreasing the peptide potency for hNa_V_1.7. Conversely, 7 analogues increased the apparent affinity for hNa_V_1.7, while only one of them had a decreased potency for hNa_V_1.6. These data illustrate that adequately mutating HwTx-IV contributes to large improvements of the peptide potency for hNa_V_1.6, while in parallel mildly improving the potency for hNa_V_1.7. This process reaches the point at which the potencies of some of the analogues are similar, in the low nanomolar range, for both channel subtypes.

## Materials and Methods

### Chemical Synthesis of HwTx-IV Analogues

**FIGURE 1:**
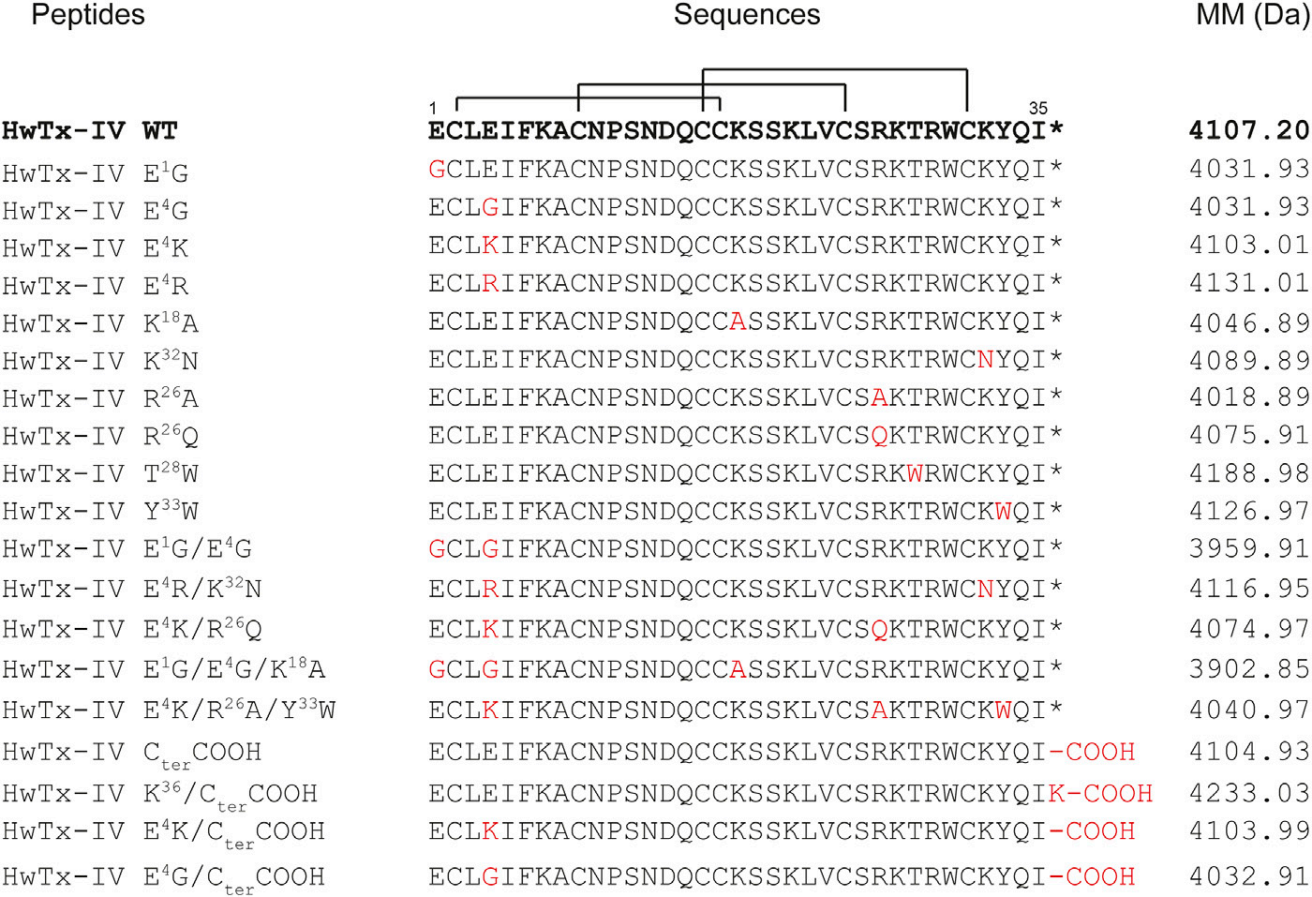
Sequence alignment, with the disulfide bond connectivity, and molecular masses of HwTx-IV and its 19 analogues. The residue substitutions in the peptide analogues are indicated in red. WT: Wild-type. MM: Molecular masses after folding/oxidation. *: C-terminal amidation.

HwTx-IV analogues were all chemically assembled and provided by Smartox Biotechnology. Briefly, assembly was done stepwise on 2-chlorotrityl chloride polystyrene resin at a 0.05 mmol or 0.1 mmol scale, using Fmoc-based Solid Phase Peptide Synthesis (SPPS) on a PTI Symphony synthesizer. 20% piperidine in DMF was used to remove the Fmoc protecting group and free amine was coupled using tenfold excess of Fmoc amino acids and HCTU/DIEA activation in NMP/DMF (3 × 15 min). Peptide de-protections and cleavages from the resin were done with TFA/H_2_O/1,3-dimethoxybenzene (DMB)/TIS/2,2′-(Ethylenedioxy)diethanethiol (DODT) 85.1/5/2.5/3.7/3.7 (vol.). They were then precipitated with cold diethyl ether. Oxidative folding of the crude linear peptides were successfully conducted at RT in the conditions optimized for HwTx-IV using a peptide concentration of 0.1 mg/ml in a 0.1 M Tris buffer at pH 8.0 containing 10% of DMSO. The amino acid sequences of HwTx-IV and its 19 analogues are shown in Figure 1, along with the molecular masses after folding/oxidation.

### Cell Cultures

CHO cells stably expressing the hNa_V_1.7 channel subtype were cultured in Dulbecco’s Modified Eagle’s Medium F-12 (DMEM/F12) supplemented with 10% fetal bovine serum, 2 mM glutamine, 200 μg/ml hygromycine B, 10 U/ml penicillin and 10 μg/ml streptomycin (Gibco, Grand Island, NY). HEK-293 cells stably expressing the hNa_V_1.6 channel subtype were cultured in Dulbecco’s Modified Eagle’s Medium (DMEM) supplemented with 10% fetal calf serum, 1 mM pyruvic acid, 4.5 g/L glucose, 2 mM glutamine, 800 μg/ml G418, 10 U/ml penicillin and 10 μg/ml streptomycin (Gibco, Grand Island, NY). All cell lines were incubated at 37°C in a 5% CO_2_ atmosphere. For electrophysiological recordings, cells were detached with trypsin, and floating single cells were diluted (∼300,000 cells/ml) in an extracellular solution containing (in mM): 140 NaCl, 4 KCl, 2 CaCl_2_, 1 MgCl_2_, 5 glucose and 10 HEPES (pH 7.4, osmolarity 298 mOsm), that was used throughout the experiments.

### Pharmacological Applications Using the Automated Patch-Clamp System

HwTx-IV analogues were investigated on CHO cells expressing hNa_v_1.7 channel and HEK-293 cells expressing hNa_V_1.6 channel using the automated patch-clamp system from Nanion (SyncroPatch 384 PE; München, Germany). Chips with single-hole and high-resistance (∼6–7 MΩ) were used for both cell lines. Voltage pulses and whole-cell recordings were achieved using the PatchControl384 v1.5.2 software (Nanion) and the Biomek v1.0 interface (Beckman Coulter). Prior to recordings, dissociated cells were shaken at 200 RPM in a cell hotel reservoir at 10°C. After cell catching, sealing, whole-cell formation, liquid application, recording, and data acquisition were all performed sequentially and automatically. The intracellular solution contained (in mM): 110 CsF, 10 CsCl, 10 NaCl, 1 MgCl_2_, 1 CaCl_2_, 10 EGTA and 10 HEPES (pH 7.2, osmolarity 280 mOsm), and the extracellular solution was as described above. Whole-cell experiments were done at −100 mV holding potential and at room temperature (18–22°C), while currents triggered at either −10 or 0 mV test potential were sampled at 20 kHz. Stimulation frequency was set at 0.2 Hz. Each HwTx-IV analogue was prepared at various concentrations in the extracellular solution, itself supplemented with 0.3% of bovine serum albumin (BSA), also added in the control solution. The peptides were distributed in 384-well microplates according to the number of compounds to be tested (generally four), the concentration range defined from the IC_50_ of wild-type HwTx-IV, and the number of cells desired for each experimental condition. Compound solutions were diluted 3 times in the patch-clamp recording well by adding 30–60 μl external solution, to reach the final reported concentration and the test volume of 90 μl. For establishing dose-response curves, the compounds were tested at a test potential of either −10 or 0 mV for 50 ms with a pulse every 5 s. Percentages of current inhibition were measured at steady-state of blockage or at the end of a 15-min application time.

### Docking of Toxin Analogues Onto hNa_V_1.7 Channel Structure

Based on the hNa_V_1.7 VSD2-Na_V_Ab chimera channel structure, obtained by cryo-electron microscopy (PDB: 6N4R), and the 3D solution structure of HwTx-IV (PDB code 1MB6), obtained by 2D ^1^H-NMR, wild-type amidated or carboxylated HwTx-IV was docked using ZDOCK v.3.0.2. Docking was used because the CryoEM structures reported in the literature lack the HwTx-IV structure in the referred PDB code (6J8G or 6J8H) (Shen et al., 2019) or because the region of interaction is not resolved according to the PDB code (6W6O) (Gao et al., 2020). Also, the earlier docking models, reported after crosslinking experiments, were not publicly available (Tzakoniati et al., 2020). Residues previously reported as implicated in HwTx-IV/hNa_V_1.7 VSD2 channel interaction (T^518^-F^761^ and S^807^-E^817^ on hNa_V_1.7 VSD2 and S^25^-I^35^ on HwTx-IV) have been used to guide docking. Only K^32^ of HwTx-IV has been selected as a conserved binding site residue for all analogues. Interactions between HwTx-IV and hNa_V_1.7 VSD2 amino acids have been analyzed using Discovery Studio (Dassault System Biovia) and the resulting 3D structures were drawn with the PyMOL software (The PyMOL Molecular Graphics System, v.2.0 Schrödinger, LLC).

## Results

### Chemical Syntheses of HwTx-IV Analogues

Overall, nineteen analogues were successfully chemically synthesized using fmoc solid-phase peptide chemistry. Proper folding and oxidation of the peptides were evaluated by checking the correspondence between theoretical *versus* experimental masses as assessed by mass spectrometry (Figure 1). Ten analogues with single amino acid substitution were produced, followed by three analogues with double amino acid substitutions, two analogues with triple amino acid substitutions, and four analogues with modifications at the C-terminus. It was remarkable that none of the analogues posed problems for the folding of the peptide (RP-HPLC data and yield calculations, not shown) in spite of the fact that this peptide folds according to an ICK motif and that some analogues harbor up to three substitutions.

### Single Amino Acid-Substituted HwTx-IV Analogues Affecting Peptide Potency Onto hNa_V_1.7

Some of the earlier SAR investigations were performed with recombinant HwTx-IV peptides that are lacking C-terminal amidation (Minassian et al., 2013; Sermadiras et al., 2013; Rahnama et al., 2017; Neff et al., 2020), an amidation that is however crucial for potency onto hNa_V_1.7 (Revell et al., 2013). Also, in some of these reports, several recombinant analogues carried additional residues at either the N- or the C-terminus, all susceptible to affect peptide potency (Minassian et al., 2013; Sermadiras et al., 2013; Neff et al., 2020). Therefore, some analogues that were deemed of interest were tested herein on hNa_V_1.7 to confirm or infirm earlier reports in more standardized conditions (normal C-terminal amidation and absence of extra-non-native amino acid residues).

**FIGURE 2:**
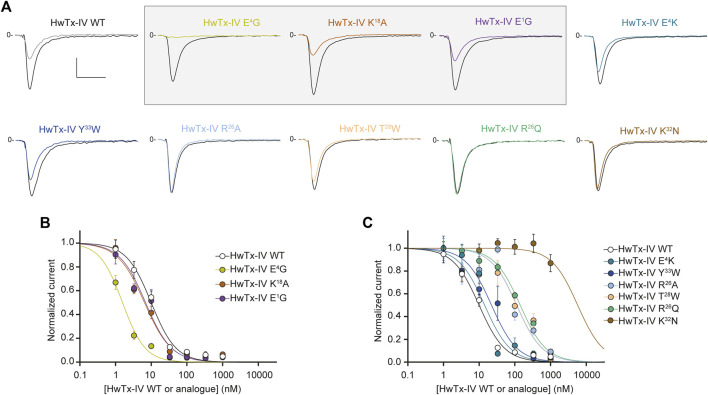
Effects of HwTx-IV and its nine mono-substituted amino acid analogues on CHO cells stably expressing the hNa_V_1.7 channel subtype. **(A)** Representative traces of current flowing through the hNa_V_1.7 subtype, recorded before (black traces) and after (coloured traces) exposure to 10 nM of the indicated peptide. Vertical and horizontal scales (applied to all traces) are 0.5 nA and 5 ms, respectively. The mono-substituted amino acid analogues more potent than HwTx-IV are highlighted by a grey rectangle. **(B)** and **(C)** Concentration-response curves of the effects of HwTx-IV (white circles) and analogues (coloured circles) on hNa_V_1.7 peak current, recorded during a −10 mV test pulse voltage. Each value, expressed relatively to that obtained before peptide application, represents the mean ± S.E.M. of data obtained from 2-8 cells. The theoretical curves correspond to data point fits with the mean IC_50_ values indicated in Table 1.

As shown in Figure 2A, we identified 3 analogues of HwTx-IV with mono-substituted amino acid residues (HwTx-IV E^1^G, HwTx-IV E^4^G, and HwTx-IV K^18^A) that possessed better potency towards the hNa_V_1.7 channel subtype. This was best exemplified by current traces illustrating the percentage of inhibition of Na^+^ inward current at 10 nM peptide analogue (upper panel). One substitution brings in a remarkable improvement in HwTx-IX potency onto hNa_V_1.7: the E^4^G mutation with an IC_50_ value which is 6.2-fold better than wild-type HwTx-IV (Figure 2B). The improvements made by the E^1^G or the K^18^A mutations were milder. All other mono-substituted analogues of HwTx-IV had decreased potencies towards hNa_V_1.7 with most of them producing almost no inhibition at all at 10 nM (Figure 2A). As shown by the average dose-response curves, the E^4^K mutation was most conservative, followed by the Y^33^W one. As expected from earlier reports (see Discussion section), the most disruptive single point mutation was K^32^N, while *R* ^26^A, *R* ^26^Q and T^28^W had milder effects on the HwTx-IV loss of potency (Figure 2C and Table 1).

**TABLE 1:** Mean ± S.E.M. of IC_50_ values (n cells) determined from the concentration-response curves of the effects of wild-type (WT) HwTx-IV and analogues on hNa_V_1.7 and hNa_V_1.6 peak currents.

	hNa_V_1.7				hNa_V_1.6			
	Mean IC_50_ (nM)	SE.M.	n	*versus* WT	Mean IC_50_ (nM)	SE.M.	n	*versus* WT
**HwTx-IV WT**	**9.9**	**1.1**	**5–7**	**1.00**	**226.6**	**1.2**	**6–10**	**1.00**
HwTx-IV E^1^G	6.9	1.1	3–7	0.70	62.4	1.2	4–7	0.28
HwTx-IV E^4^G	1.6	1.1	6–7	0.16	6.3	1.3	3	0.03
HwTx-IV E^4^K	14.2	1.2	2–8	1.43	20.9	1.3	3	0.09
HwTx-IV E^4^R	—	—	—	—	11.1	1.3	3–5	0.05
HwTx-IV K^18^A	6.4	1.1	3–7	0.65	416.2	1.3	3–7	1.84
HwTx-IV K^32^N	5752.0	1.6	4–7	581	>1,000	-	2–7	>4.4
HwTx-IV *R* ^26^A	111.7	1.2	4–7	11.28	614.2	1.2	3–6	2.71
HwTx-IV *R* ^26^Q	141.4	1.1	3–5	14.28	>1,000	-	2–8	>4.4
HwTx-IV T^28^W	114.1	1.2	4–6	11.53	—	—	—	—
HwTx-IV Y^33^W	22.2	1.2	3–7	2.24	207.2	1.3	3–9	0.91
HwTx-IV E^1^G/E^4^G	2.0	1.1	3–7	0.20	3.7	1.2	1–2	0.02
HwTx-IV E^4^R/K^32^N	>1,000	—	2–8	>101	>333	-	2–3	>1.5
HwTx-IV E^4^K/*R* ^26^Q	3.6	1.1	2–8	0.36	3.4	1.5	5	0.02
HwTx-IV E^1^G/E^4^G/K^18^A	0.8	1.1	3–7	0.08	4.9	1.2	4	0.02
HwTx-IV E^4^K/*R* ^26^A/Y^33^W	0.9	1.1	3–8	0.09	86.4	1.4	5	0.38
HwTx-IV C_ter_COOH	176.3	1.1	5–8	17.81	1,230.0	1.3	2–6	5.43
HwTx-IV K^36^/C_ter_COOH	29.0	1.2	2–6	2.93	1,334.0	1.6	2–7	5.89
HwTx-IV E^4^K/C_ter_COOH	11.9	1.1	4–7	1.20	135.7	1.3	4–10	0.60
HwTx-IV E^4^G/C_ter_COOH	14.9	1.1	2–7	1.51	55.1	1.2	5–10	0.24

Bold value highlight the wild-type peptide for a better comparison with analogues.

### Single Amino Acid-Substituted HwTx-IV Analogues Affecting Peptide Potency Onto hNa_V_1.6

**FIGURE 3:**
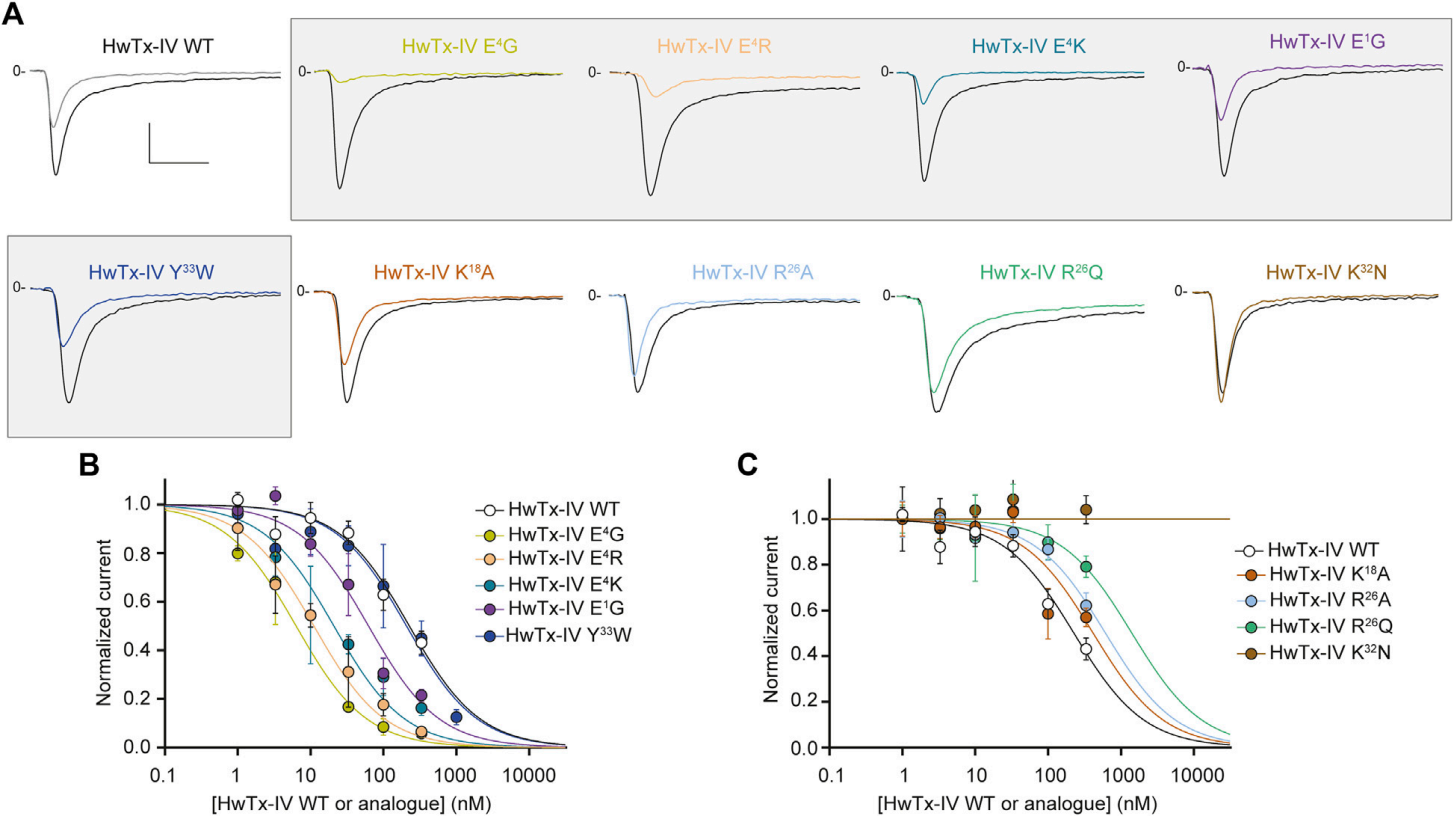
Effects of HwTx-IV and its nine mono-substituted amino acid analogues on HEK-293 cells stably expressing the hNa_V_1.6 channel subtype. **(A)** Representative traces of current flowing through the hNa_V_1.6 subtype, recorded before (black traces) and after (coloured traces) exposure to 100 nM of the indicated peptide. Vertical and horizontal scales (applied to all traces) are 0.5 nA and 5 ms, respectively. The mono-substituted amino acid analogues more potent than HwTx-IV are highlighted by grey rectangles. **(B)** and **(C)** Concentration-response curves of the effects of HwTx-IV (white circles) and analogues (coloured circles) on hNa_V_1.6 peak current, recorded during a 0-mV test-pulse voltage. Each value, expressed relatively to that obtained before peptide application, represents the mean ± S.E.M. of data obtained from 2-10 cells. The theoretical curves correspond to data point fits with the mean IC_50_ values indicated in Table 1.

**FIGURE 4:**
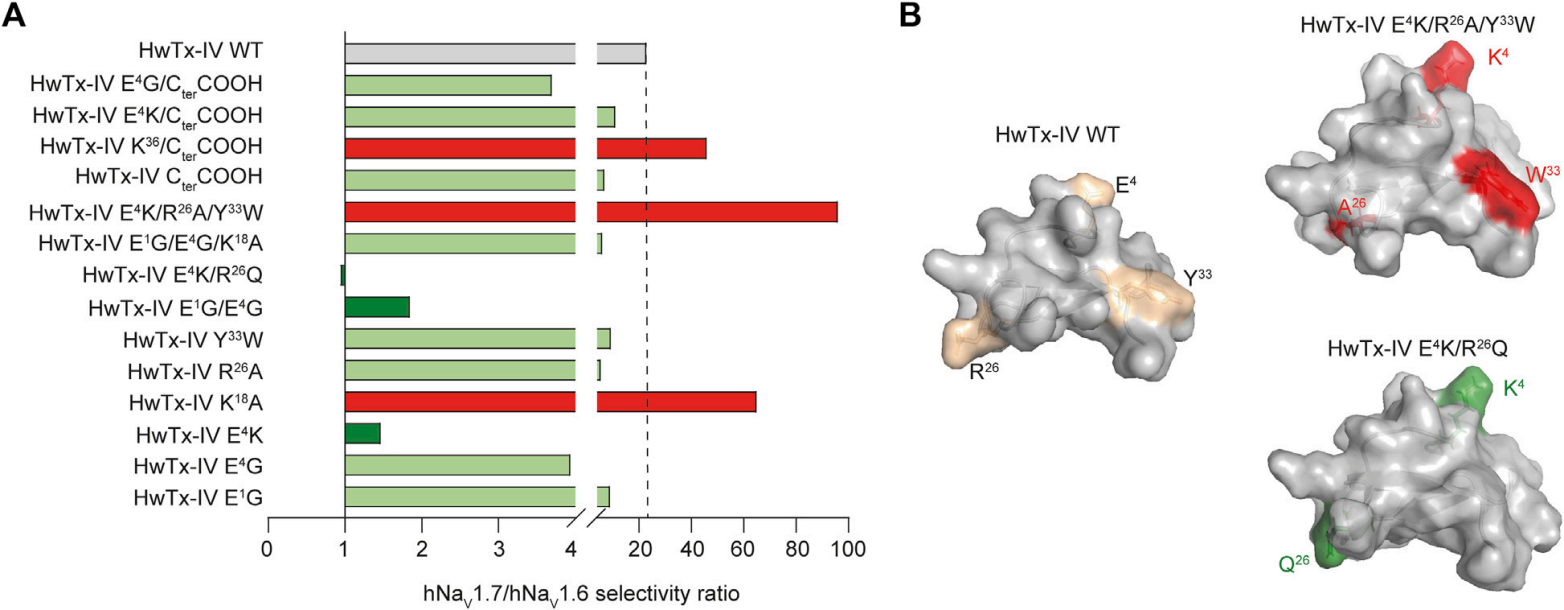
Effects of HwTx-IV and analogues on the hNa_V_1.7/hNa_V_1.6 selectivity ratio. **(A)** Histogram of hNa_V_1.7/hNa_V_1.6 selectivity ratio of HwTx-IV and analogues (dashed line: reference value for HwTx-IV WT; ratio > WT value in red and ratio < WT value in green), determined from the inverse ratio of their respective IC _50_ mean values indicated in Table 1. **(B)** Structural representation of two analogues compared to HwTx-IV WT: HwTx-IV E^4^K/*R* ^26^Q for selectivity ratio close to 1, and HwTx-IV E^4^K/*R* ^26^A/Y^33^W for enhanced selectivity ratio.

We next tested wild-type HwTx-IV along with its nine mono-substituted amino acid analogues onto the Na^+^ current carried by hNa_V_1.6 subtype. We used a concentration of 100 nM to evaluate which analogue performed better than the wild-type HwTx-IV on Na^+^ current inhibition. As shown in Figure 3A, 100 nM of HwTx-IV inhibits less than 50% of hNa_V_1.6 current, which is coherent with the average IC_50_ value of 226.6 nM (Figure 3B). As such, natively, without any sequence alteration, HwTx-IV is 22.9-fold more potent on hNa_V_1.7 than on hNa_V_1.6 channel subtype (starting selectivity ratio as detailed in Figure 4). Five out of nine mono-substituted amino acid analogues displayed better potency on hNa_V_1.6 than wild-type HwTx-IV itself: HwTx-IV E^1^G (3.6-fold better), HwTx-IV E^4^G (36-fold better), HwTx-IV E^4^K (10.8-fold better), HwTx-IV E^4^R (20.4-fold better) and HwTx-IV Y^33^W (1.1-fold better).

In contrast, 4 mono-substituted analogues of HwTx-IV displayed lower potencies for hNa_V_1.6 compared to wild-type HwTx-IV. Two analogues still affected hNa_V_1.6 with measurable potency: HwTx-IV K^18^A (1.8-fold reduction) and HwTx-IV *R* ^26^A (2.7-fold reduction). The two other analogues have a too significant shift in IC_50_ values to be measured (HwTx-IV K^32^N and HwTx-IV *R* ^26^Q, both >1 μM). In conclusion, among mono-substituted HwTx-IV analogues, the most interesting ones are those with a sequence alteration at position 4 (HwTx-IV E^4^G or HwTx-IV E^4^K) or 18 (HwTx-IV K^18^A) since these analogues led to an improved potency for hNa_V_1.7 (Figure 4 and Table 1). This improved potency of HwTx-IV K^18^A was associated to an improved selectivity *versus* hNa_V_1.6, which is likely to reduce the molecule side effects resulting from impairment of neuromuscular transmission. In contrast, HwTx-IV E^4^G and HwTx-IV E^4^K tended clearly to abolish differences between potencies for hNa_V_1.7 and hNa_V_1.6, allowing to produce pan-Na_V_ blockers.

### Combining Several Substitutions Onto HwTx-IV May Also Bring Competitive Advantages in Improving hNa_V_1.7/hNa_V_1.6 Selectivity Ratio

**FIGURE 5:**
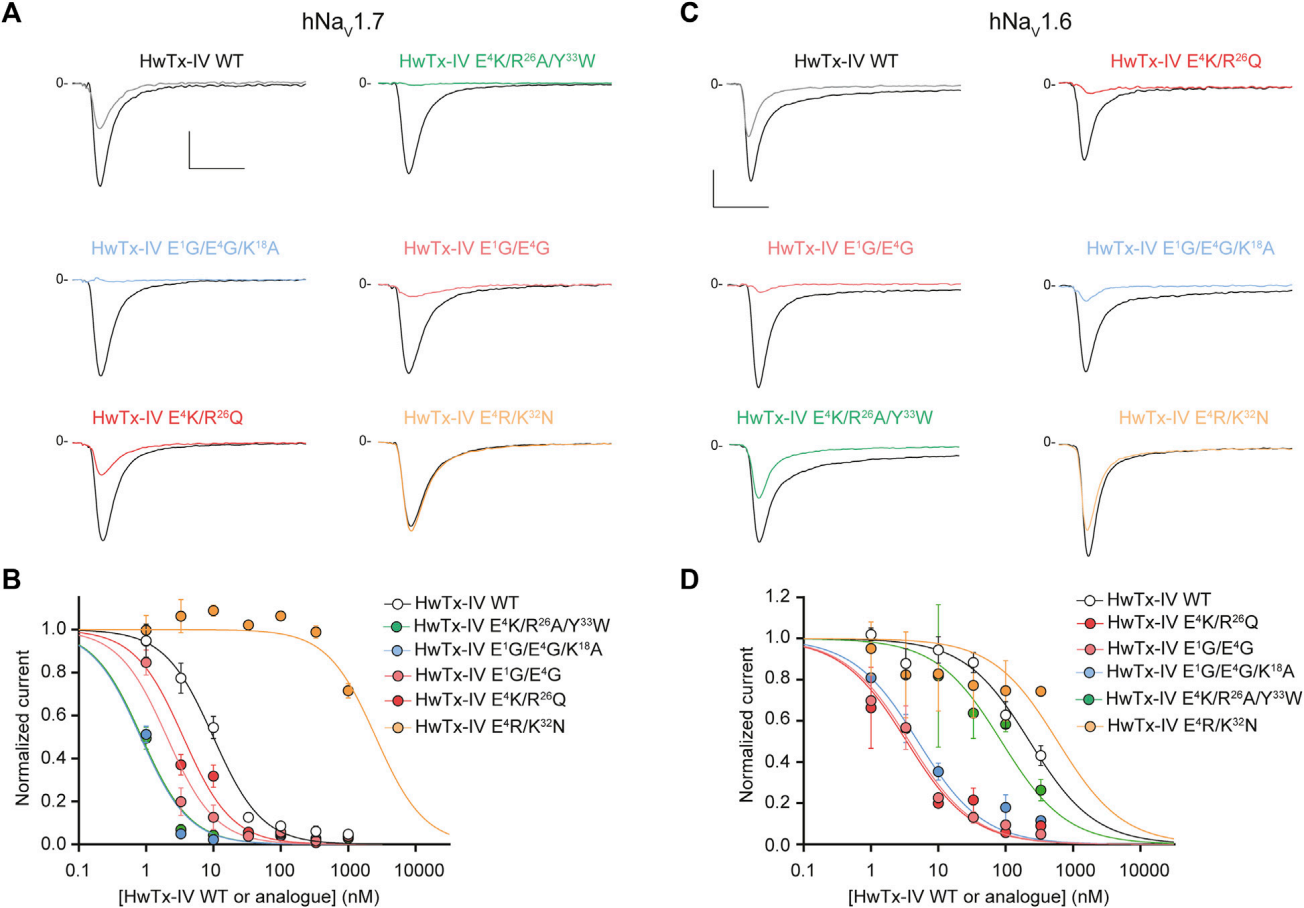
Effects of HwTx-IV and its five double- or triple-substituted amino acid analogues on CHO and HEK-293 cells stably expressing the hNa_V_1.7 **(A,B)** and hNa_V_1.6 **(C,D)** channel subtypes, respectively. (A) and (C) Representative traces of current flowing through the hNa_V_1.7 (A) or hNa_V_1.6 (C) subtype, recorded before (black traces) and after (coloured traces) exposure to 10 nM (A) or 100 nM (C) of the indicated peptide. Vertical and horizontal scales (applied to all traces) are 0.5 nA and 5 ms, respectively. (B) and (D) Concentration-response curves of the effects of HwTx-IV (white circles) and analogues (coloured circles) on hNa_V_1.7 (B) and hNa_V_1.6 (D) peak currents, recorded during −10-mV and 0-mV test-pulse voltages, respectively. Each value, expressed relatively to that obtained before peptide application, represents the mean ± S.E.M. of data obtained from 1-8 cells. The theoretical curves correspond to data point fits with the mean IC_50_ values indicated in Table 1.

We first attempted to combine two or three substitutions that individually were shown to be beneficial for the potency of HwTx-IV onto hNa_V_1.7 (E^1^G, E^4^G, K^18^A) or hNa_V_1.6 (E^1^G, E^4^G) (Figures 2A, Figure 3A). The double mutant HwTx-IV E^1^G/E^4^G produced IC_50_ values of 2.0 ± 1.1 nM for hNa_V_1.7 (Figure 5A) and 3.7 ± 1.2 nM for hNa_V_1.6 (Figure 5B). These values are more or less similar to those brought by the single E^4^G substitution, but with a slight improvement introduced by the additional E^1^G mutation in the case of the hNa_V_1.6 subtype. Adding the K^18^A substitution onto the HwTx-IV E^1^G/E^4^G mutant (HwTx-IV E^1^G/E^4^G/K^18^A) did not result in a marked benefit of the peptide potency onto both targets (Figure 5). This was expected since the effect of K^18^A substitution was similar to that of the E^1^G substitution and 4-fold lower to that of the E^4^G substitution on the hNa_V_1.7 subtype. In contrast to the E^1^G and E^4^G substitutions, it did not, by itself, drastically change HwTx-IV potency for the hNa_V_1.6 subtype (Figures 2A, 3A and Table 1). In a second attempt, we combined two substitutions that individually were, *a priori*, not favorable for potency improvements, *i.e. R* ^26^Q and E^4^K, although the latter was advantageous for hNa_V_1.6 (Figures 2B, 3).

Nevertheless, the combination of these two substitutions had some logic since they introduce compensatory charge alterations in the whole peptide sequence (loss of negative charge and gain of positive charge at position 4, the latter being compensated by the loss of positive charge at position 26). Interestingly, the double mutant HwTx-IV E^4^K/*R* ^26^Q yielded excellent potency of the peptide for both channel subtypes (Figure 5). Noteworthy, this double mutation yielded yet another HwTx-IV analogue, along with HwTx-IV E^1^G and HwTx-IV E^1^G/E^4^G, that possesses similar and high potencies (low nanomolar range) for both channel subtypes. Introducing a third substitution at position 33, which individually was quite neutral for both channel types (Figures 2B, 3A), added to those at positions 4 and 26, did not markedly modify the potency for hNa_V_1.7 but produced a 25.4-fold decrease of that for hNa_V_1.6 compared to HwTx-IV E^4^K/*R* ^26^Q (Figures 4, 5 and Table 1).

We also attempted to produce a charge compensation of the E^4^R mutation, similar to *R* ^26^Q in the HwTx-IV E^4^K/*R* ^26^Q analogue, by an analogous K^32^N mutation. As observed by the IC_50_ values (>333 nM in both cases, Figure 5), this strategy did not work because of the crucial role of K^32^ in the pharmacophore of HwTx-IV (Minassian et al., 2013; Revell et al., 2013).

### Examining the Importance of HwTx-IV C-Terminal Amidation on Peptide Potency for Both Na_V_ Subtypes

**FIGURE 6:**
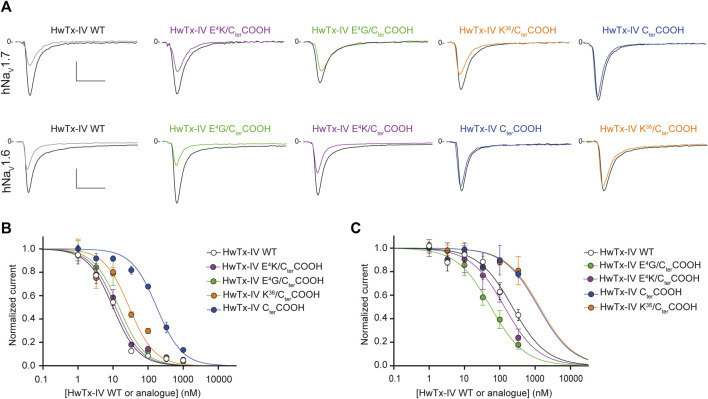
Effects of HwTx-IV and its four C-terminal-altered analogues on CHO and HEK-293 cells stably expressing the hNa_V_1.7 and hNa_V_1.6 channel subtypes, respectively. **(A)** Representative traces of current flowing through the hNa_V_1.7 (upper traces) or hNa_V_1.6 (lower traces) subtype, recorded before (black traces) and after (coloured traces) exposure to 10 nM (upper traces) or 100 nM (lower traces) of the indicated peptide. Vertical and horizontal scales (applied to all traces) are 0.5 nA and 5 ms (all traces). **(B)** and **(C)** Concentration-response curves of the effects of HwTx-IV (white circles) and analogues (coloured circles) on hNa_V_1.7 (B) and hNa_V_1.6 (C) peak currents, recorded during −10-mV and 0-mV test-pulse voltages, respectively. Each value, expressed relatively to that obtained before peptide application, represents the mean ± S.E.M. of data obtained from 1-8 cells. The theoretical curves correspond to data point fits with the mean IC_50_ values indicated in Table 1.

We first replaced the C-terminal amidated residue by a carboxylated version of the residue. As shown in Figure 6, the potency of HwTx-IV C_ter_COOH was significantly reduced on both channel subtypes (by 18-fold on hNa_V_1.7 and by 5-fold on hNa_V_1.6). These results confirm an earlier report on the role of amidation in HwTx-IV activity (Revell et al., 2013). It is likely that the consequence of amidation on HwTx-IV potency may be explained by the presence of a positive charge at the C-terminus. Indeed, the potency of HwTx-IV K^36^/C_ter_COOH, in which the peptide is still carboxylated but integrates an additional K residue, positively-charged on its side-chain, was greatly improved for the hNa_V_1.7 but not hNa_V_1.6 subtype (Figure 6). Similar results were obtained when E^4^ was mutated in order to remove the negative charge (E^4^G), or to replace the negative charge by a positive one (E^4^K) at this position. Hence, these results are reminiscent of those obtained with the deleterious *R* ^26^ mutation that also could rescue HwTx-IV potency by an additional E^4^ substitution (Figure 5). Considering the distal positioning of E^4^ relatively to *R* ^26^ or to C_ter_CONH_2_, the beneficial effect of E^4^ substitution is likely due to a rebalance in dipole moment.

## Discussion

In this manuscript, our goal was to produce HwTx-IV analogues with increased potencies for hNa_V_1.7, hNa_V_1.6, or both. Based on previous studies, we examined a number of analogues for which only a few observations were reported. Finding analogues with potent activity for hNa_V_1.6 was a particular challenge because the original hNa_V_1.7/hNa_V_1.6 selectivity ratio of 23 was largely in favor of hNa_V_1.7. This endeavor was successful, thanks to mono-, double- or triple-amino acid substitutions. Despite an excellent starting potency for hNa_V_1.7 (mean IC_50_ value of 9.9 nM, in accordance with earlier reports (Xiao et al., 2008; Revell et al., 2013; Sermadiras et al., 2013; Rahnama et al., 2017; Agwa et al., 2020)), it was still possible to further improve the activity of HwTx-IV on this channel subtype by mutations of specific positions, *i.e.* E^1^, E^4^, K^18^, E^1^/E^4^, E^4^/*R* ^26^, E^1^/E^4^/K^18^ and E^4^/*R* ^26^/Y^33^. The maximal optimization factor on potency was 11–12-fold (with the triple mutants HwTx-IV E^4^K/*R* ^26^A/Y^33^W and E^1^G/E^4^G/K^18^A). Concerning hNa_V_1.6, the challenge was easier to overcome since the starting potency of HwTx-IV was quite low for this channel subtype (mean IC_50_ value of 227 nM). The best analogues were (ranked by decreasing order of improved potency): HwTx-IV E^4^K/*R* ^26^Q, E^1^G/E^4^G, E^1^G/E^4^G/K^18^A, E^4^G, and E^4^R, all under or close to 10 nM of IC_50_ value (improvement by 20- to 67-fold), and HwTx-IV E^4^K, E^1^G, E^4^K/*R* ^26^A/Y^33^W and Y^33^W with less noticeable improvement (by 1.1- to 11-fold). Five analogues had comparable potencies (low nanomolar range) on the two channel subtypes: HwTx-IV E^4^G, E^4^K, E^1^G/E^4^G, E^4^K/*R* ^26^Q and E^1^G/E^4^G/K^18^A. These data highlight the importance of the type of amino acid at position 4 in obliterating the difference between potencies for the two channel subtypes. However, substitutions at position 4 were not all equipotent since E^4^G was more potent than E^4^K on hNa_V_1.7, possibly because G^4^ was more lipid-friendly than the charged K^4^, at least for this channel type. With regard to selectivity ratios, two observations can be made: 1) some analogues display a large increased selectivity ratio in favor of hNa_V_1.7 (K^18^A, E^4^K/*R* ^26^A/Y^33^W and K^36^/C_ter_COOH, all above 46), while keeping excellent apparent affinity on this subtype, and 2) other analogues display a significant reduction in selectivity ratio in favor of hNa_V_1.6 (E^4^K, E^1^G/E^4^G and E^4^K/*R* ^26^Q, less than 2) (Figure 4 and Table 1).

### Fitting Our Data With Earlier Reports

**FIGURE 7:**
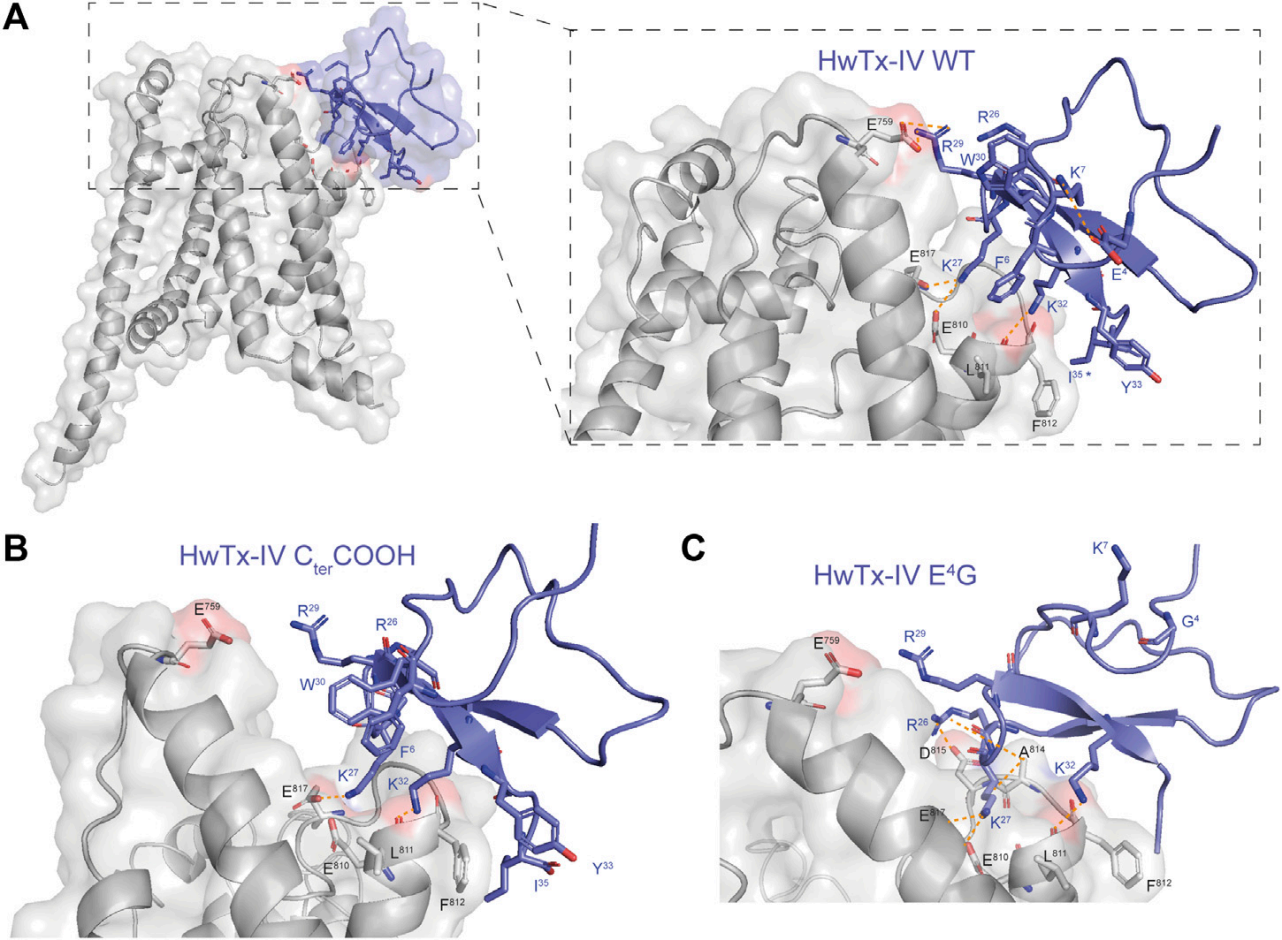
Docking of HwTx-IV analogues on hNa_V_1.7 VSD2-Na_V_Ab. **(A)** Docking of HwTx-IV WT with amidated C-terminal (C_ter_) confirming the key peptide contributions of K^27^, *R* ^29^ and K^32^. **(B)** Docking of the HwTx-IV analogue harboring a carboxylated C_ter_ shows loss of interaction through *R* ^29^ and K^27^ in accordance with a loss of potency. **(C)** Docking of the HwTx-IV E^4^G analogue with its amidated C_ter_ shows loss of electrostatic interaction between the residue at position 4 and K^7^ and larger changes in interactions with VSD2. Residues interacting with VSD2 are shown in sticks. Interactions between HwTx-IV and VSD2 residues are represented by dashed orange lines.

HwTx-IV has been the focus of several SAR studies, mainly with the aim to improve selectivity for hNa_V_1.7 *versus* other channel subtypes. Indeed, HwTx-IV suffers from toxicity issues *in vivo*, most of the symptoms being however related to an action on Na_V_1.6 (Gonçalves et al., 2018b), a subtype that was not the topic of the earlier SAR investigations. In our study, we found HwTx-IV to be quite potent on hNa_V_1.7 with a mean IC_50_ value of 9.9 nM, which compares favorably with the literature as far as the peptide was produced chemically (Sermadiras et al., 2013; Neff et al., 2020). Hence, when HwTx-IV was produced according to recombinant approaches, a significant decrease of potency was always observed (Minassian et al., 2013; Sermadiras et al., 2013; Neff et al., 2020). This decrease may be explained by two factors. The first one is simply the absence of C-terminal amidation. Indeed, in our study, the loss of C-terminal amidation results in a 18-fold decline of potency for hNa_V_1.7 of the chemically-synthesized peptide (see Figures 6A,B and Table 1). This finding is coherent with an earlier report (Revell et al., 2013). According to our docking simulations, this loss of potency should be due to a reduction of the number of interaction points with hNa_V_1.7 (Figure 7). The second one may be due to incomplete or improper folding of the peptide, which is less controlled using recombinant production than chemical synthesis. Indeed, a comparison between the potencies of amidated synthetic and carboxylated recombinant HwTx-IV shows an even more important decline of potency for hNa_V_1.7 in one study (up to 66-fold) (Sermadiras et al., 2013).

Until 2018, to our knowledge, there was no report illustrating the activity of HwTx-IV on Na_V_1.6, a tetrodotoxin (TTX)-sensitive channel subtype present in the Ranvier nodes of α-motoneurons (Rahnama et al., 2017). Since then, Gonçalves and collaborators showed that the peptide blocks this channel subtype with an IC_50_ value slightly below 100 nM (Gonçalves et al., 2018b), which is not markedly different from the value we obtained in standardized conditions. These results allow a better understanding of the mechanisms of *in vivo* motor toxicity side-effects of HwTx-IV (Liu et al., 2014b; Deuis et al., 2016). More recently, a large-scale SAR investigation was performed with the help of recombinant HwTx-IV analogues tested on both hNa_V_1.7 and hNa_V_1.2 and, occasionally, on a larger panel of other hNa_V_ subtypes, without extensively reporting on the activity of wild-type HwTx-IV on hNa_V_1.6 (Neff et al., 2020).

### Lessons From SAR Studies to Determine HwTx-IV Critical Residues for hNa_V_1.7 Block

Several objectives have been pursued in HwTx-IV SAR studies, namely: 1) defining the site of binding of the peptide onto the channel, 2) determining the peptide pharmacophore to get a clear picture of the peptide docking on the channel, 3) clarifying the relationships between the peptide and the channel lipid environment, and 4) improving both the peptide potency and selectivity. The two first aims are closely interconnected.

As stated above (see Introduction section), the blocking effect of HxTx-IV on hNa_V_1.7 is due to its binding onto the voltage-sensor of domain II of the channel subtype. As a result, HwTx-IV traps this voltage-sensor in the inward closed configuration (Xiao et al., 2008), which logically inhibits the gating currents conveyed by domain II movement (Xiao et al., 2014). More precisely, the binding occurs onto the S3-S4 linker of domain II. Amino acid residues D^816^ and E^818^ of hNa_V_1.7 are involved in this binding since mutating these residues reduces the peptide potency by over 60-fold (Xiao et al., 2008; Xiao et al., 2010). Conversely, mutations in hNa_V_1.4 (N^655^D, Q^657^E) or hNa_V_1.5 (R^812^D, S^814^E), aimed at instating hNa_V_1.7 residues, were shown to produce a gain of HwTx-IV potency on these channel subtypes. A larger scale investigation refined the pictures of hNa_V_1.7 channel residues involved in binding HwTx-IV, and identified a total of five amino acids, one belonging to the S1-S2 linker (E^753^) and the four remaining ones being part of the S3-S4 linker (E^811^, L^814^, D^816^ and E^818^) (Xiao et al., 2011). This cluster of 5 residues defines an EELDE motif (herein defined as E_1_E_2_L_3_D_4_E_5_ for convenience), and thus suggests that the interaction largely relies on electrostatic interactions. It is worth noting that HwTx-IV acts in an odd manner on the voltage-sensor of domain II since no clear voltage-dependent effect of the toxin has been reported in the literature, except maybe for very strong depolarizing pulses meant to favor toxin dissociation (Gonçalves et al., 2018b). However, SAR studies may potentially reveal new HwTx-IV analogues displaying voltage-dependent effect and, therefore, it would be of interest to check the voltage-dependence or lack thereof of the best toxin analogues.

To get a hint of how the toxin interacts with its binding site on hNa_V_1.7, several initiatives were launched, including 1) complete SAR investigation of the contribution of HwTx-IV residues, 2) cryo-EM analyses and 3) the use of photocrosslinking probes derived from HwTx-IV.

Concerning the SAR studies on HwTx-IV, the emerging picture is that the couple of residues, W^30^ and K^32^, are required to preserve peptide activity onto hNa_V_1.7 (Minassian et al., 2013; Revell et al., 2013; Neff et al., 2020). Modest reduction in potencies (<10-fold) are observed for F^6^A, K^18^A, *R* ^26^A, K^27^A and Y^33^A, all other substitutions being neutral (L^3^A, S^9^A, P^11^A, S^12^A, D^14^A, L^22^A, S^25^A) or modestly beneficial (E^1^A, E^4^A, K^7^A, N^10^A, Q^12^A, S^19^A, S^20^A, K^21^A, V^23^A, T^28^A, *R* ^29^A, Q^34^A and I^35^A) (Revell et al., 2013). These conclusions are relatively well supported by the data of Minassian and collaborators, although these authors use mainly recombinant HwTx-IV, lacking C-terminal amidation and possessing some extra residues in the sequence (Minassian et al., 2013).

For the cryo-EM investigation, the authors replaced the S3-S4 linker of the NaChBac sequence by the one of hNa_V_1.7, and illustrated the interaction of HwTx-IV with this chimera channel (Gao et al., 2020). They concluded that K^27^ is located close to E_2_, that K^32^ interacts with E_2_, and that Y^33^ forms hydrogen bonds with the backbone groups of L_3_ and an adjacent A residue. Globally, positive residues of HwTx-IV *R* ^26^, K^27^ and K^32^ are in close vicinity of the channel, whereas the hydrophobic residues I^5^, F^6^ and W^30^ are immersed in the lipid bilayer. However, chimera approaches may not perfectly reconstitute the wild-type HwTx-IV binding site. For instance, the K^32^A mutation in HwTx-IV barely impacts peptide effect on the chimera channel (Gao et al., 2020) while, in our study, a similar K^32^N mutation prevents the HwTx-IV-induced block of hNa_V_1.7 (see Figures 2A,C).

The model of interaction was refined by an elegant study that used photocrosslinking probes derived from HwTx-IV and the full-length hNa_V_1.7 (Tzakoniati et al., 2020). L-photomethionine was introduced in HwTx-IV sequence to replace one of 6 residues (I^5^, K^7^, K^21^, K^27^, *R* ^29^, I^35^) deemed proximal to other crucial residues involved in toxin interaction. Among these 6 new analogues, only 2 photoprobes, with substitutions at position 27 or 29, crosslinked to hNa_V_1.7 with high efficiency: photoprobe 27 to the peptide ^808^SLVE^811^ that belongs to the S3 helix, and photoprobe 29 to the motif ^758^TEEF^761^ of the S1-S2 loop. These data support the concept that K^27^ of HwTx-IV interacts with E_2_ of the E_1_E_2_L_3_D_4_E_5_ motif and *R* ^29^ with a negatively charged residue of S1-S2 loop (most likely E^759^) that differs from E_1_. In this way, they contradict the docking model of Minassian and collaborators that suggested an interaction between K^27^ and E_5_ rather than E_2_ (Minassian et al., 2013). The refined docking model supports the idea that F^6^, W^30^ and Y^33^ interact with the lipid bilayer. The crucial K^32^ residue would also interact with E_2_ of the binding motif (Figure 7). According to a summary of this docking, HwTx-IV I^35^ would also interact with L_3_. The fact that the *R* ^29^A mutation hardly affects HwTx-IV potency for hNa_V_1.7 (Revell et al., 2013) would indicate that HwTx-IV interaction with the S1-S2 loop has little functional implication for the mechanism of channel block.

How do these data fit with what we know about the toxin/lipid interactions? Part of the conclusions drawn about the definition of HwTx-IV pharmacophore was complexified by the fact that the peptide, besides binding to the targeted channel, also requires partitioning into the lipid membrane for the access to the binding site. This has led to the concept of a three-way interaction for the mechanism of action of the toxin (Agwa et al., 2017), which complicates the design of new analogues as exemplified by the case of protoxin II (Montnach et al., 2021). From the various docking models or Cryo-EM structure previously published (Minassian et al., 2013; Gao et al., 2020; Neff et al., 2020; Tzakoniati et al., 2020), several residues of HwTx-IV are susceptible to directly interact with lipids (I^5^, F^6^, *R* ^29^, W^30^, Y^33^). By mutating non-essential residues for the interaction with hNa_V_1.7, it was shown that the combined mutagenesis of E^1^G, E^4^G, F^6^W and Y^33^W produces an HwTx-IV analogue with increased ability to bind membrane lipids, a property that in turns explains the increased potency of this analogue for hNa_V_1.7 (Agwa et al., 2017). In this mutated analogue, two of the residues are suspected to interact directly with lipids (W^30^ and Y^33^), whereas the two other residues may hamper toxin partitioning into the lipid bilayer because of the negative charges associated to E residues. In wild-type HwTx-IV, the E^4^ residue forms a salt bridge with K^7^, possibly stabilizing the peptide fold. Mutation of E^4^ to G^4^ appears to increase the potency for hNa_V_1.7 by improving permeability into the membrane (Klint et al., 2015) and the interaction surface of the peptide with the channel (Figure 7). If in addition the authors mutate a non-essential positively charged residue, *R* ^26^A, a further improvement in lipid affinity is observed, also accompanied by an additional improvement in toxin potency for hNa_V_1.7 (Agwa et al., 2020). The lesson from this work is that it is possible to enhance lipid partitioning of a toxin either directly by acting on residues that interact with lipids or indirectly by altering the global hydrophobicity profile of the peptide.

It is therefore clear from SAR and docking studies that there are three ways to alter potency and selectivity of HwTx-IV, namely by 1) mutating residues involved in binding onto the channel itself, 2) substituting residues involved in peptide/lipid interactions, and 3) indirectly playing on the surface charge or hydrophobicity profile of the peptide. By intervening on one or several of these factors, it is possible to modify peptide selectivity. Earlier efforts in that direction were performed by altering the relative potency of HwTx-IV for hNa_V_1.7 and hNa_V_1.2. For instance, the *R* ^29^A mutation was shown to increase potency for hNa_V_1.7 but to considerably reduce the one for hNa_V_1.2 (Minassian et al., 2013). This was possibly due to a weakened electrostatic interaction with the EELNE motif of hNa_V_1.2. It was also found that the triple mutant E^1^G/E^4^G/Y^33^W (but as recombinant protein, *i.e.* without C-terminal amidation) has an improved IC_50_ value for hNa_V_1.7, but without increased activity for either hNa_V_1.1, hNa_V_1.2, hNa_V_1.3, hNa_V_1.5 or hNa_V_1.6 (Rahnama et al., 2017). The analogue with 5 mutations (E^1^G/E^4^G/F^6^W/*R* ^26^A/Y^3^W), not only possessed a better potency for hNa_V_1.7, but also an improved selectivity profile over hNa_V_1.2 (27 times more selective) (Agwa et al., 2020). The same analogue produced a hNa_V_1.7/hNa_V_1.6 selectivity ratio of 11.

### The Pros and Cons of SAR Studies Using HwTx-IV as Template for Selectivity Alterations

The first issue to be discussed is without any doubts linked to the strategy employed to perform the SAR investigation. With an economical perspective, it appears logic to search for analogues that are produced by recombinant means as production costs are lower and yields higher. This method was chosen by several private actors (MedImmune, Janssen Research and Development) but not all (Genentech). Clearly, a comparison of our data testing HwTx-IV analogues onto hNa_V_1.7 illustrates that the presence or not of the C-terminal amidation has important consequences on the interpretation of the SAR data. Interesting differences were noted. For instance, a substitution of T^28^ was considered as beneficial for HwTx-IV potency towards hNa_V_1.7 in its recombinant form (Revell et al., 2013), while in our hands it was clearly detrimental. Also, while for both hNa_V_1.7 and hNa_V_1.6 channel subtypes, a HwTx-IV analogue with a carboxylated C-terminus is synonym of loss of potency, there are interesting compensatory mutations capable to restore peptide potency. Part of the beneficial effect of C-terminal amidation could be reproduced by the introduction of an extra K^36^ residue, even if the C-terminus was still carboxylated (HwTx-IV K^36^/C_ter_COOH), suggesting that the amine function of the lateral chain could substitute favorably to the C-terminal amidation. Gain of potency for hNa_V_1.7 has been observed earlier if an extra Gly residue was incorporated at position 36 along with a C-terminal amidation (Sermadiras et al., 2013). The gain of potency we observed with HwTx-IV K^36^/C_ter_COOH was true for the hNa_V_1.7 target but not for the hNa_V_1.6 channel subtype, suggesting that the C-terminal amidation is a function of interest to create differences in selectivity between the two channel subtypes. These observations also question the “true” selectivity profiles published with recombinant HwTx-IV. Interestingly, mutation of E^4^ also largely compensated for the loss of potency induced by the carboxylated C-terminus sequence. This time, the compensation at work was valid for both channel subtypes, suggesting that all efforts to build recombinant analogues with increased potencies for Na_V_ channel subtypes should incorporate an E^4^ mutation.

The question that arises now is how far one can expect to go with efforts dedicated at improving selectivity for a given Na_V_ subtype? In the work we launched, the task was challenging since the beginning of the project because the crucial E_1_E_2_L_3_D_4_E motif is conserved in hNa_V_1.6. As a reminder, this motif becomes E_1_E_2_L_3_N_4_E_5_ in hNa_V_1.2, a channel subtype that was used as template for earlier SAR studies aimed at altering peptide selectivity. We are aware that these earlier efforts did not necessarily constitute the best guidelines for producing analogues differentially affecting hNa_V_1.7 and hNa_V_1.6 for two reasons: 1) the hNa_V_1.6 binding motif differs from the hNa_V_1.2 motif, and 2) the SAR studies were all performed with recombinant peptides that in addition possess several extra residues at their C-terminus (Neff et al., 2020). Nevertheless, positions that differentially affected the relative hNa_V_1.7/hNa_V_1.2 selectivity ratio when mutated were F^6^, K^18^, *R* ^26^, K^27^ and I^35^ (Minassian et al., 2013). In contrast to two reports (Minassian et al., 2013; Neff et al., 2020), we found that the K^26^ mutation preferentially lowers potency for hNa_V_1.7. More coherent was the fact that the K^18^A mutation improves potency for hNa_V_1.7 while slightly reducing it for hNa_V_1.6, although less drastically than for hNa_V_1.2 (Minassian et al., 2013). In our hands, the most interesting positions tested to differentially affect the selectivity between hNa_V_1.7 and hNa_V_1.6 were E^1^, E^4^, *R* ^26^ or the combinations E^1^/E^4^, E^4^/*R* ^26^ and E^1^/E^4^/K^18^. For obvious economic reasons, this was not an as exhaustive work as wished to test all possible positions of HwTx-IV sequence and perform a full comparison with the work of Neff and collaborators (Neff et al., 2020). Nevertheless, several conclusions could be reached. First, it is possible to further increase the potency of HwTx-IV for hNa_V_1.7 in spite of an excellent starting affinity. However, the optimization remains in the range of 10-fold maximum, suggesting that there are limitations reached maybe because of the complexity in the docking to the channel combined to the need for partitioning into the lipid membrane. Second, it is clearly possible to optimize potency for hNa_V_1.6 in spite of similarities in the target-binding site, and the extent of this optimization is greater for this subtype than for hNa_V_1.7. Thirdly, there must be differences somewhere in the docking onto hNa_V_1.6 compared to hNa_V_1.7 because some analogues clearly affected the selectivity ratios. One in particular looks as a promising hNa_V_1.7 target (HwTx-IV E^4^K/*R* ^26^A/Y^33^W) as it both improves the potency on this channel type and decreases the one on hNa_V_1.6, which should lead to decreased side effects.

## Conclusion

Overall, this work demonstrates that it is possible to create a number of new HwTx-IV analogues with increased potencies for both channel subtypes up to the point of equal potencies on hNa_V_1.7 and hNa_V_1.6. However, it was never possible to create an inversion of the selectivity ratio with a loss of potency towards hNa_V_1.7 combined with nanomolar affinity for hNa_V_1.6. For such a result, we suggest that it is better to start SAR investigation on a toxin that initially has better affinity towards Na_V_1.6.

## Data Availability

The raw data supporting the conclusions of this article will be made available by the authors, without undue reservation.
